# Psychoactive Drugs in the Management of Post Traumatic Stress Disorder: A Promising New Horizon

**DOI:** 10.7759/cureus.25235

**Published:** 2022-05-23

**Authors:** Kawther N Elsouri, Sahand Kalhori, Diego Colunge, Grant Grabarczyk, George Hanna, Cassidy Carrasco, Andy Aleman Espino, Andres Francisco, Bradley Borosky, Bassem Bekheit, Maha Ighanifard, Andrea A Astudillo, Michelle Demory Beckler

**Affiliations:** 1 Osteopathic Medicine, Nova Southeastern University Dr. Kiran C. Patel College of Osteopathic Medicine, Fort Lauderdale, USA; 2 Microbiology and Immunology, Nova Southeastern University Dr. Kiran C. Patel College of Allopathic Medicine, Fort Lauderdale, USA

**Keywords:** psychoactive drug, psilocybin, mdma, ketamine, psychedelic assisted psychotherapy, ptsd, anxiety disorder, mental health, post traumatic stress disorder, psychoactive drugs

## Abstract

Post-traumatic stress disorder (PTSD) is an anxiety disorder that often presents after exposure to a traumatic, life-threatening event. Experiencing a traumatic event is not rare, with inciting incidents ranging from being burglarized to politically motivated genocide. While traditional psychopharmacology and psychotherapy are the mainstays of the treatment of PTSD currently, psychoactive drugs (otherwise known as psychedelics) are being explored for their novel role in the treatment of PTSD patients. Psychoactive drugs such as MDMA, ketamine, and psilocybin have been shown to specifically target and decrease fear and anxiety pathways in the brain. These unique properties hold the potential to be utilized in addressing symptoms of trauma in those with refractory or treatment-resistant PTSD. Historically, federal and state laws have restricted research into how psychoactive drugs can be used to treat mental illness due to the widespread belief that these drugs present more harm than benefit. However, the current shift in public opinion on psychedelics has propelled research to look into the benefits of these drugs for patients with mental illness. This article aims to discuss the mechanisms of how MDMA, ketamine, and psilocybin work in the PTSD brain, as well as their beneficial role in treatment.

## Introduction and background

Post-traumatic stress disorder (PTSD) is a severe, often chronic, and debilitating condition, which can develop following exposure to a traumatic event involving actual or threatened injury [1]. According to the most updated version of the Diagnostic Statistic Manual of Psychiatry [1], a traumatic stressor can include exposure to actual or threatened death, serious injury, or sexual violence by directly experiencing the event, witnessing the event as it occurred to others, learning that the event happened to a close family member or friend, or experiencing repeated exposure to aversive details of traumatic events. Symptoms of the disorder last longer than one month and include, but are not limited to, intrusive memories or dreams, dissociative reactions, intense or prolonged psychological distress, negative alterations to cognition and mood associated with the trauma, and marked alteration in arousal and reactivity associated with the event [1]. The lifetime prevalence of PTSD ranges from approximately 6-9% in national samples of the general adult population in the United States and Canada [2-5]. Higher rates of PTSD have been found in population subgroups in the United States compared to the general US population, including Native Americans living on reservations and refugees from countries where traumatic stress is endemic [6-8]. PTSD is also prevalent in asylum populations, which has shown to be detrimental and resulting in failure of passing asylum interviews [9]. COVID-19 has severely impacted the mental health of people around the world, most notably the elderly. Since the beginning of the pandemic, there has been an escalation of depression, suicide, anxiety, and PTSD amongst the elderly population [10].

**Figure 1:**
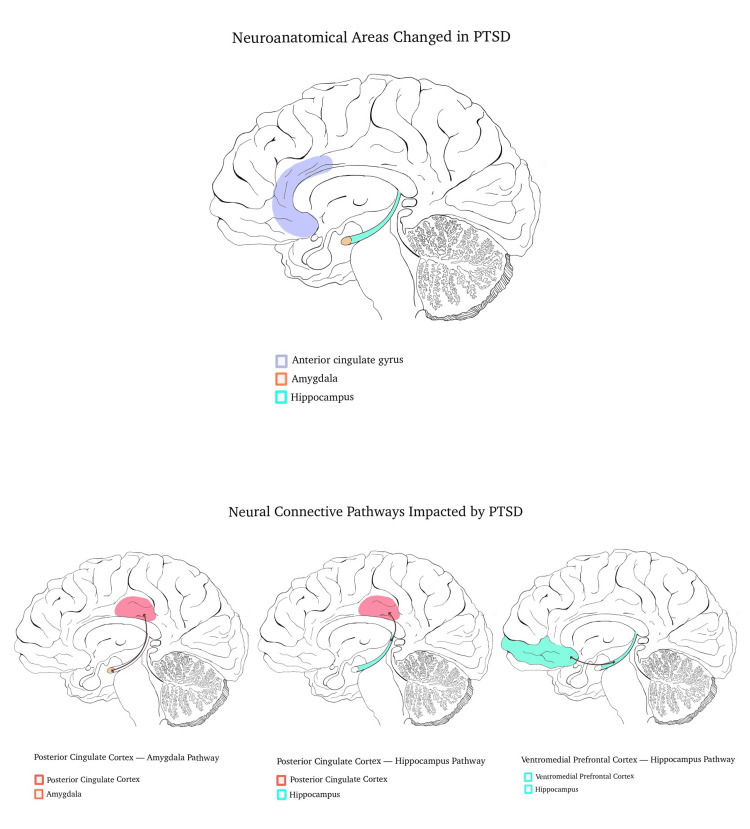
Neuroanatomical Areas and Neural Connective Pathways Associated with PTSD **original images**

While much of the pathophysiology of PTSD is complex and not fully elucidated, research findings are accruing. Studies using MRI scans have shown a decreased volume of the hippocampus, left amygdala, and anterior cingulate cortex in patients with PTSD compared to matched controls [11, 12] (Figure 1). Other reports have demonstrated increased central norepinephrine levels with down-regulated central adrenergic receptors, chronically decreased glucocorticoid levels, with up-regulation of their cognate receptors, and hemispheric lateralization in which there is a relative failure of left hemisphere function [13]. In addition to neuronal and receptor changes seen in PTSD, functional neuronal connectivity changes are being examined as key aspects of symptomatology seen in PTSD. Using functional magnetic resonance imagining (fMRI) researchers have found that PTSD patients who had a hyperarousal response to traumatic events had significantly lower pathway activation in the anterior cingulate gyrus, medial prefrontal cortex, and thalamus compared to participants who did not meet the criteria for PTSD [14]. In addition, the distinct flashbacks and dissociative responses were seen in PTSD might be attributed to abnormal functional connectivity among regions in the brain involved in different responses to trauma-related stimuli [14]. 

Similar to functional connectivity and neuroanatomical changes (Figure 1), large-scale genetic studies have shown that PTSD is a highly polygenic phenotype that is likely influenced by thousands of loci across the genome [15, 16]. Previous exposure to trauma increases the risk of developing PTSD with subsequent traumatic events [17]. The mechanism by which the sensitization to subsequent traumatic events after previous exposure to trauma, is unclear. PTSD is more prevalent amongst females than males across the lifespan [1]. Some of the increased risks have been attributable to greater likelihood of exposure to sexual traumatic events, such as rape or other forms of interpersonal violence. 

PTSD is a chronic condition, with only one-third of patients recovering at one-year follow up and one-third still symptomatic 10 years after exposure to the initiating trauma [18]. Individuals with one or more PTSD symptoms are more likely to experience higher rates of social, occupational, and physical disability, have poorer social supports, and impaired functioning across social, interpersonal, developmental, educational, and physical health domains compared to controls [19]. PTSD can increase the risk for attempted suicide [20,21]. In women with symptoms of depression, it is associated with nearly four times greater risk of death compared to women with no PTSD or depression [22]. 

Current psychotherapy techniques used for the treatment of PTSD have the goal of decreasing intrusive thoughts and images, phobic avoidance, pathological hyperarousal, hypervigilance, irritability and anger, and depression. These treatments tend to be exposure-based modalities that utilize trauma-related cues and imagery to address and resolve the fear response [23]. While psychotherapy has proven to decrease PTSD symptoms in many patients, there is still a large percentage who do not benefit from treatment. 

Due to the refractory nature of PTSD, new pharmacological regimens are being explored in an attempt to find a more effective treatment [24]. Various psychoactive drugs are being studied for their possible therapeutic effects when used in conjunction with traditional psychotherapy. Among the drugs being researched for this purpose are psychedelics including ketamine, psilocybin, and MDMA. These psychoactive drugs have been shown to be capable of facilitating structural and functional reorganization of neural circuits to produce positive behavioral effects, which may be of great benefit in many mental health disorders [25]. While there is a lot of research addressing the therapeutic and curative abilities of psychedelic drugs, not all have found that there is much benefit to the utilization of these drugs. A systematic review published in 2020 found that the quality of evidence supporting the use of ketamine as a stand-alone treatment for comorbid PTSD with depression was “very low” [26]. However, when ketamine was used in combination with psychotherapy, improvement of symptoms was sustained for approximately one month [26]. Additionally, results from this systematic review show the evidence for MDMA in combination with psychotherapy was graded as “moderate” [26]. In this review, we discuss current front-line therapies and aim to highlight mechanisms of action for psychedelics and their associated beneficial effects in the treatment of PTSD. 

## Review

Current frontline treatment 

For most adults, the recommended first-line treatment for PTSD is trauma-focused psychotherapy. Evidence-based psychotherapies include exposure therapy and a combination of exposure and cognitive-based therapy (trauma-focused cognitive behavioral therapy [TF-CBT]) [27, 28]. Exposure therapy involves exposing the patient to the anxiety source or its context without the intention to cause any danger. Doing so is thought to help patients overcome their anxiety or distress. The goal of TF-CBT is to help those who have experienced trauma learn how to manage difficult emotions in a healthier way. Patients are shown how perceptions may be distorted and are given the tools to redesign those perceptions.

Pharmacotherapy is encouraged for patients who prefer medication to psychotherapy, or for patients in which TF-CBT is not accessible. Randomized clinical trials have found that patients with PTSD experience reduced symptoms of sleep disturbances, night sweats, fatigue, and difficulty with memory or concentration when treated with a Selective Serotonergic Reuptake Inhibitor (SSRI) compared to placebo [29]. Although SSRIs are associated with an overall response rate of approximately 60% in patients with PTSD, only 20-30% of patients achieve complete remission [30]. Additionally, randomized trials have found venlafaxine, a serotonin norepinephrine reuptake inhibitor (SNRI), to be efficacious in PTSD [28]. In a study of extended-release venlafaxine, the response rate was 78% and the remission rate was 40% in patients with PTSD [31]. In addition to SSRIs and SNRIs, benzodiazepines (BZDs) have been utilized for patients with PTSD [32]. However, due to their anxiolytic, sedative, hypnotic, and skeletal muscle-relaxing effects, BZDs have become widely abused amongst this population [32]. Studies have found that this trend of misuse has only increased with the recent COVID-19 global pandemic [32].

Comparative randomized trials of psychotherapy versus pharmacotherapy have found mixed results. Evidence exists which highlights the advantage of symptom improvement with prolonged exposure therapy compared to pharmacotherapy alone [33]. Of note, when comparing the two traditional treatments, a significant improvement in symptom reduction was achieved when allowing patients to select their treatment modality. Patient adherence to treatment was improved when patients received their preferred treatment [34]. 

The neural pathways involved in PTSD and targeted with CBT are of importance to further understand the disease and develop a better treatment. In a 2005 study, Farrow et al. evaluated whether CBT would change the activation of these neural areas involved in the physiology of social cognition (the ability to forgive and empathize) in individuals with PTSD [35, 36]. The participants underwent fMRI before and after treatment to evaluate for social cognition changes. Thirteen participants who fulfilled the criteria of the DSM-IV for PTSD participated in the trial. The main finding was that after the treatment, patients experienced symptom improvement accompanied by increased brain activity in areas that were previously related to social cognition [36]. Specifically, there was an increase in the activation of the left medial temporal gyrus in response to the paradigm of empathy. The same process occurred with the posterior cingulate gyrus, which had its activation increased in response to the condition of forgiveness after the treatment. From this study, Farrow et al. concluded that CBT can promote changes in neural circuitry [35]. 

Due to the population of poor responders to traditional treatment, it is imperative that alternative treatments be studied. Recent estimates suggest that up to 50% of individuals with PTSD who engage in treatment fail to respond adequately [29]. The current PTSD treatment guidelines can be effective for some patients, but there is room for improvement with regard to response rate and remission rate. Psychedelic medications may provide a possible solution to this treatment response problem based on published research.

MDMA

Prior to 1985, ± 3,4-methylenedioxymethamphetamine (MDMA) was readily used as adjunctive therapy with psychotherapy for PTSD. In 1985, the Drug Enforcement Administration (DEA) classified MDMA as a Schedule I drug, thereby limiting its use for PTSD. Accordingly, the ability of US-based researchers to investigate its beneficial effects on PTSD declined [37]. Amongst the most well-supported studies that have been published on MDMA’s effect on PTSD patients look at the correlation between neuroanatomical changes of various brain structures and the mechanisms of action. These changes seem to be associated with MDMA’s effects on patients in regard to symptom relief, posttraumatic growth, and sleep quality. 

MDMA works as an indirect serotonin agonist by binding and inhibiting the serotonin reuptake transporter [37]. In addition, it increases the release of other monoamine neurotransmitters such as dopamine and noradrenaline [38]. MDMA appears to impact users by decreasing their reactivity to negative stimuli such as threats while enhancing feelings of empathy, openness, and euphoria [39] through the release of both dopamine and noradrenaline. These effects seemingly target and address many of the fears of negative stimuli faced by PTSD patients. This led to the FDA recently granting a breakthrough therapy designation to MDMA, allowing for larger, well-controlled clinical trials to commence [40]. 

In clinical trials, patients suffering from PTSD have demonstrated that MDMA is effective, well-tolerated, and has no major serious long-term adverse effects [41]. In 2014, an fMRI study highlighted the importance of the clinical effects that MDMA can have on PTSD patients [38]. The results showed decreased left amygdala activity and increased anterior cingulate cortex activation in the participants who took MDMA. This finding is critical because imaging studies on patients with PTSD exposed to a fear paradigm find that the left amygdala is activated while the anterior cingulate cortex is deactivated [38]. These two structures help regulate emotions such as fear, which plays a big role in PTSD. Additionally, the MDMA cohort reported fewer negative feelings associated with their worst memories compared to controls/placebo groups [38].

Another similar study looked at participants' brains with functional MRI while they listened to two three-minute blocks of their own trauma script [42]. Following the MDMA treatment, the same trauma scripts were associated with increased activity in the medial and orbital prefrontal cortices, as well as mid-cingulate and supplementary motor areas [42]. These findings suggest that part of MDMA’s therapeutic utility is increased signaling in the prefrontal limbic circuitry, showing increased engagement with the trauma script in PTSD patients [42].

Of patients suffering from PTSD, veterans of the armed services and first responders do not appear to respond very well to the current front-line treatments such as CBT and SSRIs [43]. Several studies have aimed to study the efficacy of MDMA-assisted psychotherapy in these populations. A randomized, double-blind, dose-response clinical trial for military veterans or first responders with diagnosed chronic PTSD according to the Clinician-Administered PTSD Scale (CAPS-IV), showed that increased doses of MDMA in conjunction with psychotherapy decreased PTSD symptom severity one month after treatment [39, 43]. PTSD short-term symptom severity was also significantly decreased in participants that were treated with an initial low dose and a subsequent higher dose one month later, suggesting that high-dose administration of MDMA is effective at relieving PTSD symptoms [43]. A similar study evaluated the effects of multiple doses of MDMA on both short- and long-term outcomes in PTSD patients. PTSD symptoms were assessed using CAPS-IV scoring one to two months following the last active MDMA session and 12 months after the last active session [41]. The majority of the patients in the trial reported that their symptoms were reduced after one to two months and remained lower 12 months following the treatment [41]. 

An additional aspect of PTSD where MDMA could potentially assist patients is posttraumatic growth (PTG), which consists of the positive changes a patient sees in oneself as a result of the trauma [44]. A recent study showed that participants with PTSD with a score higher than 50 on the CAPS-IV scale and who were unresponsive to pharmacological and psychotherapy interventions, showed higher levels of posttraumatic growth and a larger reduction of symptoms compared to controls at both one month and one year post one high dose of MDMA treatment [44]. Furthermore, two-thirds of the MDMA-treated participants ultimately failed to meet the criteria for PTSD at the conclusion of the study, strongly supporting the notion that MDMA can be a vital tool in helping PTSD patients overcome their disease [44]. 

Poor sleep quality is among the most common and distressing symptoms seen in PTSD [45]. Sleep problems can interfere with the brain's ability to process emotions such as trauma, resulting in a longer recovery process following a traumatic event [45]. Given the euphoric feelings associated with MDMA treatment, MDMA treatment was examined to determine whether it might help improve patient sleep quality. Ponte et al. used the Pittsburgh Sleep Quality Index (PSQI) to assess sleep quality in patients from baseline, primary endpoint, treatment exit, and 12-month follow-up [46]. At all points where patients' sleep quality was measured after either MDMA or placebo treatment, the MDMA group showed increased sleep quality compared to the placebo group reported at the 12-month follow-up [47].

Ketamine

Ketamine is a non-competitive glutamate N-methyl-D-glutamate receptor antagonist, with a lower affinity for serotonin, dopamine, opioid, and other receptors. Ketamine was approved by the FDA as an anesthetic agent in 1970 [47]. Ketamine’s analgesic effects are accomplished by preventing central sensitization in dorsal horn neurons, which prevents the transmission of pain signals via the spinal cord [48].

In patients with PTSD, the use of ketamine has been proposed due to its ability to repair synaptic connectivity [49]. Chronic stress from PTSD can cause neural atrophy and decrease the number of synapses within cortical and limbic circuits, which are associated with the regulation of mood, cognition, and behavior [49]. The dominant form of synapses in these circuits is linked with glutamate [49]. Stress can compromise the integrity of signaling via glutamate synapses in several ways, including reducing signaling via brain-derived neurotrophic factor (BDNF) and impairing its downstream intracellular signaling [49]. Under the effects of chronic stress, there are reductions in the amount of α-amino-3-hydroxy-5-methyl-4-isoxazolepriopionic acid (AMPA) glutamate receptors in the synapse, reduced size of dendritic spines, loss of dendritic spines that support synaptic connectivity, and even loss of larger dendritic elements resulting in decreased dendritic complexity [49]. 

Ketamine is thought to work by enhancing synaptic connectivity and by increasing dendritic spines in the apical dendrites of pyramidal neurons in superficial cortical layers, thereby reversing the effects of stress caused by PTSD [49]. While research regarding the detailed mechanism of ketamine in PTSD is limited, research on the outcome and benefits of ketamine for these patients is abundant. 

Early case reports using ketamine in treatment-resistant PTSD demonstrated rapid and sustained improvement in patient symptoms, including a reduction in flashbacks, with subanesthetic doses that were well tolerated with no adverse effects [50]. For example, an early case study of a military veteran with treatment-resistant PTSD showed a 56% improvement of PTSD symptoms maintained for 15 days after a single infusion of a subanesthetic dose of ketamine. The ketamine infusion was well-tolerated, and no adverse effects were reported [50]. Later, a larger cohort study consisting of participants diagnosed with PTSD, showed that a single infusion of 0.5 mg/kg of ketamine over 40 min led to significant improvement in chronic PTSD symptoms 24 hours post-infusion compared to midazolam, a standardized anxiolytic [51]. Dissociative symptoms and physical adverse effects during infusion were noted as being transient and well-tolerated [51]. 

In 2021, a randomized-controlled clinical trial showed that repeated intravenous ketamine infusions demonstrated sustained improvement in chronic PTSD patients [52]. The participants suffered from severe-to-chronic PTSD determined by the CAPS-5 (Clinician-Administered PTSD Scale for DSM-5), MADRS (Montgomery-Asberg Depression Rating Scale), and other parameters such as duration of PTSD and source of primary trauma (i.e. sexual assault, molestation, physical assault, combat exposure) [52]. The participants experienced PTSD for a median duration of 15 years, half of whom were taking concomitant psychotropic medications. Within the first 24 hours post-infusion, the ketamine group showed marked improvements in three of the four PTSD symptoms: intrusions, avoidance, and negative alterations in cognition and mood, compared to the control [52]. This improvement lasted for a median of 27.5 days. Participants undergoing ketamine treatment also showed a significant reduction in comorbid depressive symptoms. Repeated ketamine infusions were shown to be both safe and generally well-tolerated among study participants [52]. This is a promising result due to it being the first randomized clinical trial to prove both safety and efficacy with repetitive ketamine infusions to combat chronic PTSD.

A concern for the use of ketamine has been whether adverse effects of treatment can lead to neurocognitive changes in participants. In a population of 16 veterans suffering from comorbid PTSD and treatment-resistant depression (TRD), six IV infusions of 0.5 mg/kg ketamine over a 12-day period were administered with the intention of monitoring neurocognitive function. The study showed no reduction in cognitive performance and in fact, cognitive assessments were predictive of a positive response to ketamine treatment [53]. With these results, Albott et al. proceeded to determine the therapeutic response and durability of six infusions of ketamine to veterans with comorbid PTSD and TRD in a different study [54]. In comparison to single-infusion regimens, this regimen resulted in a longer period of symptom reduction. In this sample population of participants in PTSD remission, 80% of participants maintained this response for two weeks, which is more than three times the rate of responders for single infusion treatments. The median time to PTSD relapse was 41 days which is indicative of the longevity of a repetitive ketamine infusion regimen [54]. However, treatment was shown to be safe and tolerable across all participants, with transient increases in dissociative symptoms peaking immediately following cessation of infusion treatment and complete resolution of symptoms two hours later [54]. 

Psilocybin

While psilocybin was originally studied for the treatment of psychiatric illness in the mid-1900s, the FDA ban and class I scheduling in the Controlled Substance Act of 1970 halted research. It wasn’t until 1992 that the FDA allowed for research with psychedelics [55]. Even with the relatively new lease to research psychedelics, there is little investigation into psilocybin as an adjunct treatment to psychotherapy for PTSD. 

The mechanism of action of psilocin, the active metabolite of psilocybin, is the agonism of the 5HT-2A, 5HT-2C, and 5HT-1A receptors. 5HT-2A receptor is the main route by which psilocybin causes its hallucinogenic effects [56]. Agonism of the 5HT-2A receptor is shown to enhance the inhibitory effect of the visual and prefrontal cortex on the amygdala [57]. Downregulation of the amygdala in response to fearful stimuli is the mechanism that is suggested to be related to the mood-elevation caused by the use of psilocybin [57]. 

Despite the lack of studies on the efficacy of psilocybin in the treatment of PTSD, evidence collected before the controlled substance ban showed that it is safe to use with few long-term adverse events [54]. In addition, a single-arm, open-label pilot study showed that psilocybin treatment for demoralization in AIDS patients showed few acute adverse events [58]. In a double-blind study by Grob et al. in 2011, 12 participants with advanced cancer diagnoses were dosed with 0.2mg/kg of psilocybin or a niacin placebo [59]. The low dosage was tolerated well amongst this population [59]. Ross et al. and Griffiths et al. both completed double-blinded studies with psilocybin to treat depression and anxiety in participants with life-threatening cancer diagnoses, with 29 and 51 participants respectively [60,61]. Neither study reported any serious adverse events due to the administration of psilocybin to patients [60,61]. Minor side effects reported by these aforementioned trials include headache, nausea, elevated systolic and diastolic pressures, acute anxiety, and fatigue. Overall, the side effects of psilocybin administration are relatively mild and acute indicating that there is low potential for long-term adverse effects from psilocybin. 

With these current limitations in mind, psilocybin may still be a promising treatment for PTSD. While the exact pathophysiology of PTSD is not fully elucidated, it is thought that a dysregulated fear response is one facet of the disease state [26]. fMRI studies of participants’ brains after dosing psilocybin show decreased cerebral blood flow (CBF) and decreased reactivity in the amygdala [56, 62]. Decreased reactivity of the amygdala is associated with a decreased fear response. In conjunction with psychotherapy, psilocybin may help attenuate the negative reaction to stimuli that patients can be exposed to during psychotherapy. Psilocybin has also been noted by Catlow et al. to cause fear extinction in animal models, which can further diminish the fear response to traumatic stimuli experienced by PTSD patients [63]. Kometer et al. discovered that psilocybin enhanced positive mood, potentially reducing negative thoughts in PTSD patients that experience comorbid depression [64]. However, Abdallah et al. also noted that the default mode network (DMN) is hypoactive and weakly interconnected in those with PTSD [26]. A hypoactive DMN is associated with symptoms such as avoidance, dissociation, and intrusive thoughts which are observed in PTSD [26]. Carhart-Harris et al. published an fMRI study in 2012 that performed blood-oxygen level-dependent fMRI on 15 participants injected with either 2mg psilocybin in 10mL saline or a 10mL saline placebo [65]. The results show psilocybin induces lower levels of CBF in the DMN, among several other regions of the brain [65]. The effects of reduced CBF in the DMN induced by psilocybin need further investigation into how it will affect the hypoactive DMN resting-state observed in PTSD.

While psilocybin has shown potential for the treatment of PTSD, the gap in research does not provide enough evidence to indicate whether psilocybin as a co-therapy with psychotherapy, is useful in the treatment of PTSD. Additionally, the current legal stance on psilocybin creates several hurdles for researchers to overcome. The time and capital required to train therapists as well as acquire a suitable stock of psilocybin can be prohibitive to studies that are not well funded [55]. For more substantial research to be completed, changes in the legal status of psilocybin are likely needed to allow researchers better access to the compound. Further pilot and feasibility studies should investigate psilocybin effectiveness in populations affected by PTSD and PTSD with comorbid MDD.

## Conclusions

The psychopharmacology of psychedelics has been extensively studied over the past 20 years. For decades, research has focused on the pernicious effects of psychedelics while disregarding the clinical benefits these drugs may serve. The more recent shift in policies and public opinion provides a new horizon for the use of psychoactive drugs in managing PTSD. The prevalence rate for PTSD ranges between 6-8% amongst the general public and significantly higher amongst veterans. Effects of PTSD can involve intrusive thoughts and images, phobic avoidance, pathological hyperarousal, hypervigilance, irritability and anger, and depression. Traditional treatments utilize trauma-focused cognitive behavioral therapy and/or pharmacotherapy to modify and treat PTSD behavior. About two-thirds of patients have shown a response rate to traditional treatment with pharmacotherapy but 40% or less have shown remission. This is the basis on which other outlets such as MDMA, ketamine, and psilocybin are being researched.

MDMA with psychotherapy has shown a lasting significant decrease in chronic PTSD symptoms in veterans and first responders when administered at increasing dosages over a therapeutic period. Using fMRI and a trauma script, MDMA shows increased prefrontal limbic signaling, meaning that fear is more easily regulated by PTSD patients. Ketamine use shows promise in PTSD patients as well as comorbid treatment-resistant depression. More studies need to be performed to show the resolution and remission in comparison to a placebo. While not many studies have clearly elucidated the mechanism of psilocybin, the drug has shown to decrease blood flow to the amygdala, indicating a decreased fear response. This leaves room for future research to explore the mechanism of this psychedelic for the treatment of PTSD. 

Overall, psychedelics show promise as being therapeutic and curative options for PTSD patients. Future research should consider and address the methodological and conceptual limitations of currently published findings. It should aim to assess the depth by which these psychedelics impact the neural connectivity and neuroanatomy of patients with PTSD. Additionally, future research should study and analyze the long-term effects of these treatments. 

## References

[1] American Psychiatric Association (2013). Diagnostic And Statistical Manual Of Mental Disorders, Fifth Edition.

[2] Kautz M, Charney DS, Murrough JW (2017). Neuropeptide Y, resilience, and PTSD therapeutics. Neurosci Lett.

[3] Kessler RC, Berglund P, Demler O, Jin R, Merikangas KR, Walters EE (2005). Lifetime prevalence and age-of-onset distributions of DSM-IV disorders in the National Comorbidity Survey Replication. Arch Gen Psychiatry.

[4] Van Ameringen M, Mancini C, Patterson B, Boyle MH (2008). Post-traumatic stress disorder in Canada. CNS Neurosci Ther.

[5] Koenen KC, Ratanatharathorn A, Ng L (2017). Posttraumatic stress disorder in the World Mental Health Surveys. Psychol Med.

[6] Goldstein RB, Smith SM, Chou SP (2016). The epidemiology of DSM-5 posttraumatic stress disorder in the United States: results from the National Epidemiologic Survey on Alcohol and Related Conditions-III. Soc Psychiatry Psychiatr Epidemiol.

[7] Beals J, Manson SM, Whitesell NR, Spicer P, Novins DK, Mitchell CM (2005). Prevalence of DSM-IV disorders and attendant help-seeking in 2 American Indian reservation populations. Arch Gen Psychiatry.

[8] Kisely S, Alichniewicz KK, Black EB, Siskind D, Spurling G, Toombs M (2017). The prevalence of depression and anxiety disorders in indigenous people of the Americas: A systematic review and meta-analysis. J Psychiatr Res.

[9] Sarangi A, Deleon S, Baronia R (2021). Severe post-traumatic disorder leading to failure of passing asylum interview—a case report. Middle East Curr Psychiatry.

[10] Sarangi A, Javed S, Karki K, Kaushal A (2021). COVID-19-associated PTSD in the elderly—lessons learned for the next global pandemic. Middle East Curr Psychiatry.

[11] Marshall GN, Schell TL, Elliott MN, Berthold SM, Chun CA (2005). Mental health of Cambodian refugees 2 decades after resettlement in the United States. JAMA.

[12] Bremner JD, Randall P, Scott TM (1995). MRI-based measurement of hippocampal volume in patients with combat-related posttraumatic stress disorder. Am J Psychiatry.

[13] Karl A, Schaefer M, Malta LS, Dörfel D, Rohleder N, Werner A (2006). A meta-analysis of structural brain abnormalities in PTSD. Neurosci Biobehav Rev.

[14] van der Kolk BA (1997). The psychobiology of posttraumatic stress disorder. J Clin Psychiatry.

[15] Lanius RA, Williamson PC, Densmore M, Boksman K, Neufeld RW, Gati JS, Menon RS (2004). The nature of traumatic memories: a 4-T FMRI functional connectivity analysis. Am J Psychiatry.

[16] Duncan LE, Cooper BN, Shen H (2018). Robust findings from 25 years of PTSD genetics research. Curr Psychiatry Rep.

[17] Gelernter J, Sun N, Polimanti R (2019). Genome-wide association study of post-traumatic stress disorder reexperiencing symptoms in &gt;165,000 US veterans. Nat Neurosci.

[18] Breslau N, Chilcoat HD, Kessler RC, Davis GC (1999). Previous exposure to trauma and PTSD effects of subsequent trauma: results from the Detroit Area Survey of Trauma. Am J Psychiatry.

[19] Kessler RC, Sonnega A, Bromet E, Hughes M, Nelson CB (1995). Posttraumatic stress disorder in the National Comorbidity Survey. Arch Gen Psychiatry.

[20] Solomon SD, Davidson JR (1997). Trauma: prevalence, impairment, service use, and cost. J Clin Psychiatry.

[21] Wilcox HC, Storr CL, Breslau N (2009). Posttraumatic stress disorder and suicide attempts in a community sample of urban american young adults. Arch Gen Psychiatry.

[22] Bernal M, Haro JM, Bernert S (2007). Risk factors for suicidality in Europe: results from the ESEMED study. J Affect Disord.

[23] Roberts AL, Kubzansky LD, Chibnik LB, Rimm EB, Koenen KC (2020). Association of posttraumatic stress and depressive symptoms with mortality in women. JAMA Netw Open.

[24] Krediet E, Bostoen T, Breeksema J, van Schagen A, Passie T, Vermetten E (2020). Reviewing the potential of psychedelics for the treatment of PTSD. Int J Neuropsychopharmacol.

[25] Olson DE (2018). Psychoplastogens: a promising class of plasticity-promoting neurotherapeutics. J Exp Neurosci.

[26] Abdallah CG, Averill LA, Akiki TJ (2019). The neurobiology and pharmacotherapy of posttraumatic stress disorder. Annu Rev Pharmacol Toxicol.

[27] Bisson J, Andrew M (2007). Psychological treatment of post-traumatic stress disorder (PTSD). Cochrane Database Syst Rev.

[28] Coventry PA, Meader N, Melton H (2020). Psychological and pharmacological interventions for posttraumatic stress disorder and comorbid mental health problems following complex traumatic events: Systematic review and component network meta-analysis. PLoS Med.

[29] Resick PA, Wachen JS, Dondanville KA (2017). Effect of group vs individual cognitive processing therapy in active-duty military seeking treatment for posttraumatic stress disorder: A randomized clinical trial. JAMA Psychiatry.

[30] Berger W, Mendlowicz MV, Marques-Portella C, Kinrys G, Fontenelle LF, Marmar CR, Figueira I (2009). Pharmacologic alternatives to antidepressants in posttraumatic stress disorder: a systematic review. Prog Neuropsychopharmacol Biol Psychiatry.

[31] Davidson J, Baldwin D, Stein DJ (2006). Treatment of posttraumatic stress disorder with venlafaxine extended release: a 6-month randomized controlled trial. Arch Gen Psychiatry.

[32] Sarangi A, McMahon T, Gude J (2021). Benzodiazepine misuse: an epidemic within a pandemic. Cureus.

[33] Rauch SA, Kim HM, Powell C (2019). Efficacy of prolonged exposure therapy, sertraline hydrochloride, and their combination among combat veterans with posttraumatic stress disorder: a randomized clinical trial. JAMA Psychiatry.

[34] Zoellner LA, Roy-Byrne PP, Mavissakalian M, Feeny NC (2019). Doubly randomized preference trial of prolonged exposure versus sertraline for treatment of PTSD. Am J Psychiatry.

[35] Farrow TF, Hunter MD, Wilkinson ID (2005). Quantifiable change in functional brain response to empathic and forgivability judgments with resolution of posttraumatic stress disorder. Psychiatry Res.

[36] Farrow TF, Zheng Y, Wilkinson ID (2001). Investigating the functional anatomy of empathy and forgiveness. Neuroreport.

[37] Amoroso T (2015). The psychopharmacology of ±3,4 methylenedioxymethamphetamine and its role in the treatment of posttraumatic stress disorder. J Psychoactive Drugs.

[38] Feduccia AA, Mithoefer MC (2018). MDMA-assisted psychotherapy for PTSD: Are memory reconsolidation and fear extinction underlying mechanisms?. Prog Neuropsychopharmacol Biol Psychiatry.

[39] Bershad AK, Miller MA, de Wit H (2017). MDMA does not alter responses to the Trier Social Stress Test in humans. Psychopharmacology (Berl).

[40] Mithoefer M, Mithoefer A, Feduccia A (2019). S.08.06 MDMA-Assisted psychotherapy for PTSD: A promising novel experimental treatment moving into phase 3 trials with FDA Breakthrough therapy designation. Eur Neuropsychopharmacol.

[41] Jerome L, Feduccia AA, Wang JB (2020). Long-term follow-up outcomes of MDMA-assisted psychotherapy for treatment of PTSD: a longitudinal pooled analysis of six phase 2 trials. Psychopharmacology (Berl).

[42] Mithoefer OJ (2015). The effects of MDMA on brain reactivity to personalized trauma scripts in patients with PTSD: A pilot study. Biol Psychiatry.

[43] Mithoefer MC, Mithoefer AT, Feduccia AA (2018). 3,4-methylenedioxymethamphetamine (MDMA)-assisted psychotherapy for post-traumatic stress disorder in military veterans, firefighters, and police officers: a randomised, double-blind, dose-response, phase 2 clinical trial. Lancet Psychiatry.

[44] Gorman I, Belser AB, Jerome L (2020). Posttraumatic growth after MDMA‐assisted psychotherapy for posttraumatic stress disorder. J Trauma Stress.

[45] Pacheco D (2022). Sleep Foundation. How post-traumatic stress disorder affects sleep. https://www.sleepfoundation.org/mental-health/ptsd-and-sleep.

[46] Ponte L, Jerome L, Hamilton S, Mithoefer MC, Yazar-Klosinski BB, Vermetten E, Feduccia AA (2021). Sleep quality improvements after MDMA-assisted psychotherapy for the treatment of posttraumatic stress disorder. J Trauma Stress.

[47] Feder A, Costi S, Rutter SB (2021). A randomized controlled trial of repeated ketamine administration for chronic posttraumatic stress disorder. Am J Psychiatry.

[48] Schnoebel R, Wolff M, Peters SC (2005). Ketamine impairs excitability in superficial dorsal horn neurones by blocking sodium and voltage-gated potassium currents. Br J Pharmacol.

[49] Krystal JH, Abdallah CG, Averill LA (2017). Synaptic loss and the pathophysiology of PTSD: implications for ketamine as a prototype novel therapeutic. Curr Psychiatry Rep.

[50] Abdallah CG, Averill LA, Krystal JH (2015). Ketamine as a promising prototype for a new generation of rapid-acting antidepressants. Ann N Y Acad Sci.

[51] Feder A, Parides MK, Murrough JW (2014). Efficacy of intravenous ketamine for treatment of chronic posttraumatic stress disorder: a randomized clinical trial. JAMA Psychiatry.

[52] Cristina Albott, Kelvin Lim, Miriam Forbes (2017). Neurocognitive effects of repeated ketamine infusions in co-occurring posttraumatic stress disorder and treatment-resistant depression. Biol Psychiatry.

[53] Albott CS, Lim KO, Forbes MK (2018). Efficacy, safety, and durability of repeated ketamine infusions for comorbid posttraumatic stress disorder and treatment-resistant depression. J Clin Psychiatry.

[54] Bird CI, Modlin NL, Rucker JJ (2021). Psilocybin and MDMA for the treatment of trauma-related psychopathology. Int Rev Psychiatry.

[55] Nutt D (2019). Psychedelic drugs-a new era in psychiatry?. Dialogues Clin Neurosci.

[56] Tylš F, Páleníček T, Horáček J (2014). Psilocybin--summary of knowledge and new perspectives. Eur Neuropsychopharmacol.

[57] Kraehenmann R, Schmidt A, Friston K, Preller KH, Seifritz E, Vollenweider FX (2016). The mixed serotonin receptor agonist psilocybin reduces threat-induced modulation of amygdala connectivity. Neuroimage Clin.

[58] Anderson BT, Danforth A, Daroff PR (2020). Psilocybin-assisted group therapy for demoralized older long-term AIDS survivor men: An open-label safety and feasibility pilot study. EClinicalMedicine.

[59] Grob CS, Danforth AL, Chopra GS (2011). Pilot study of psilocybin treatment for anxiety in patients with advanced-stage cancer. Arch Gen Psychiatry.

[60] Griffiths RR, Johnson MW, Carducci MA (2016). Psilocybin produces substantial and sustained decreases in depression and anxiety in patients with life-threatening cancer: A randomized double-blind trial. J Psychopharmacol.

[61] Ross S, Bossis A, Guss J (2016). Rapid and sustained symptom reduction following psilocybin treatment for anxiety and depression in patients with life-threatening cancer: a randomized controlled trial. J Psychopharmacol.

[62] Carhart-Harris RL, Roseman L, Bolstridge M (2017). Psilocybin for treatment-resistant depression: fMRI-measured brain mechanisms. Sci Rep.

[63] Catlow B. J., Song S., Paredes D. A. Effects of psilocybin on hippocampal neurogenesis and extinction of trace fear conditioning. Experimental Brain Research, 228, 481-491. https://doi.org/10.1007/s00221-013-3579-0.

[64] Kometer M., Schmidt A., Bachmann R. Psilocybin biases facial recognition, goal-directed behavior, and mood state toward positive relative to negative emotions through different serotonergic subreceptors. Biological Psychiatry.

[65] Carhart-Harris RL, Erritzoe D, Williams T (2012). Neural correlates of the psychedelic state as determined by fMRI studies with psilocybin. Proc Natl Acad Sci U S A.

